# Convalescence plasma treatment of COVID-19: results from a prematurely terminated randomized controlled open-label study in Southern Sweden

**DOI:** 10.1186/s13104-021-05847-7

**Published:** 2021-12-04

**Authors:** Karin Holm, Maria N. Lundgren, Jens Kjeldsen-Kragh, Oskar Ljungquist, Blenda Böttiger, Christian Wikén, Jonas Öberg, Nils Fernström, Ebba Rosendal, Anna K. Överby, Julia Wigren Byström, Mattias Forsell, Mona Landin-Olsson, Magnus Rasmussen

**Affiliations:** 1 Department of Infectious diseases, Skåne University Hospital, Lund, Sweden; 2 Department of Clinical Immunology and Transfusion Medicine, University and Regional Laboratories, Region Skåne, Sweden; 3 Clinical Infection Medicine, Department of Translational Medicine, Lund University, Malmö, Sweden; 4 Department of Clinical Microbiology, University and Regional Laboratories, Region Skåne, Sweden; 5 Department of Clinical Microbiology, Umeå University, Umeå, Sweden; 6 Department of Endocrinology, Skåne University Hospital, Lund, Sweden

**Keywords:** SARS-CoV2, COVID-19, Convalescent plasma, Oxygen therapy, Desaturation

## Abstract

**Objective:**

Convalescent plasma has been tried as therapy for various viral infections. Early observational studies of convalescent plasma treatment for hospitalized COVID-19 patients were promising, but randomized controlled studies were lacking at the time. The objective of this study was to investigate if convalescent plasma is beneficial to hospitalized patients with COVID-19.

**Results:**

Hospitalized patients with confirmed COVID-19 and an oxygen saturation below 94% were randomized 1:1 to receive convalescent plasma in addition to standard of care or standard of care only. The primary outcome was number of days of oxygen treatment to keep saturation above 93% within 28 days from inclusion. The study was prematurely terminated when thirty-one of 100 intended patients had been included. The median time of oxygen treatment among survivors was 11 days (IQR 6–15) for the convalescent plasma group and 7 days (IQR 5–9) for the standard of care group (*p* = 0.4, median difference -4). Two patients in the convalescent plasma group and three patients in the standard of care group died (*p* = 0.64, OR 0.49, 95% CI 0.08–2.79). Thus no significant differences were observed between the groups.

*Trial registration* ClinicalTrials NCT04600440, retrospectively registered Oct 23, 2020.

**Supplementary Information:**

The online version contains supplementary material available at 10.1186/s13104-021-05847-7.

## Introduction

The Severe Acute Respiratory Syndrome Coronavirus 2 (SARS-CoV-2) has caused a pandemic with coronavirus disease-19 (COVID-19). At the beginning of the pandemic several therapies were tried as antiviral drugs against SARS-CoV-2 involving chloroquine, azithromycin and ribavirin without success.

Infusion of convalescence plasma (CCP) containing specific neutralizing antibodies to SARS-CoV-2 has potential antiviral effects. Various mechanisms have been suggested as responsible for the therapeutic effect of CCP such as virus neutralization and immunomodulation [1]. Previous experiences from treatment with CCP of influenza and Ebola and the other severe corona virus infections SARS in 2003 and MERS in 2012 were promising [2–7], and inspired attempts to evaluate the treatment of COVID-19 with CCP. Early reports supported that CCP is well tolerated [8] and several observational studies indicated a possible beneficial effect of the addition of CCP to standard of care (SOC) [9–11].

In order to further examine if CCP could be beneficial as treatment for COVID-19 we conducted a randomized controlled trial of CCP as addition to SOC in patients hospitalized with COVID-19. Our hypothesis was that the median time of oxygen need to keep oxygen saturation above 93% would be significantly shorter in the CCP group than in the SOC group. The study was prematurely terminated after an interim analysis that was carried out due to increasing evidence that this treatment modality was ineffective [12, 13].

## Main text

### Methods

#### Trial design

This was an open-label randomized superiority trial performed in the Skåne University Hospital in Lund and Helsingborg Hospital, both in the Skåne Region in Southern Sweden, between June 2020 and January 2021 (ClinicalTrials NCT04600440, date of registration Oct 23, 2020). The Swedish Ethical Review Authority approved the study (reference number #2020-01744, 2020-03595). Written informed consent was required from donors and patients on inclusion. This study adheres to the CONSORT guidelines.

### Participants

Patients admitted to the hospital with a nasopharyngeal swab positive for SARS-CoV-2 in RT-PCR no later than 4 days prior to inclusion and a need for supplemental oxygen treatment to keep peripheral oxygen saturation > 93% were eligible for inclusion. Written informed consent was required from the patients on inclusion. Exclusion criteria were age below 18, a habitual oxygen saturation below 94%, inability to give informed consent and severe immunosuppression.

### Randomization and intervention

Patients eligible for inclusion were enrolled and randomized by a study physician (KH, OL, CW, JO, MR), 1:1 using the electronic software REDCap to either receive SOC or SOC with 200–250 mL of CCP administered intravenously during 30 min on three consecutive days. Blocks of ten patients were used for randomization. The intended number of participants in each group was 50. At the time of designing the study, there was inadequate data on expected time of oxygen need and the expected variation between patients. Therefore, our decision to include 100 patients was based on feasibility to complete the study rather than power estimates. An open label rather than double blinded design was used since we could not within reasonable time arrange a placebo infusion.

### Preparing of plasma

Donors of convalescent plasma were recruited among individuals diagnosed with COVID-19 by a positive RT-PCR-test at the regional microbiology laboratory. All donors had mild to moderate disease and fulfilled the national blood donor selection criteria. Plasma was collected at least 2 weeks after the complete resolution of clinical symptoms. Only male donors were eligible in order to reduce the risk of transfusion-associated acute lung injury in the recipient due to HLA- and/or granulocyte-antibodies that are present in the plasma of some female donors. Donor IgG antibodies against SARS-CoV-2 spike protein were detected by in-house ELISA followed by the measurement of titers of neutralizing antibodies (NtAbs) by a modified microneutralization assay on Vero E6 cells (See Additional file 1: Methods) at the Department of Clinical Microbiology, Umeå University, Sweden. A titer of at least 1:40 was required. After signed informed consent, plasmapheresis was carried out in accordance with standard protocols collecting an amount of 550–650 mL plasma that was subsequently divided into 2–3 plasma units, frozen and stored at -40 ℃ in the blood bank. In total, 19 donors provided 47 units of convalescent plasma for the study. Donors had NtAbs titers between 1:40 and 1:1160 with a median value of 1:116. Some donors with declining antibody titers during the study period were excluded from further donations.

### Monitoring

Patients were monitored once daily for adverse events, blood pressure, oxygen saturation, heart and respiratory rates and temperature. An adverse event was any adverse symptom or clinical sign that was not considered as an obvious symptom of COVID-19. Death, increased respiratory failure and abnormal laboratory tests were monitored separately and not included in adverse event monitoring. The type of respiratory assistance (oxygen therapy, oxygen therapy by high flow nasal cannula (HFNC) or non-invasive ventilation (NIV), or ventilator assistance was noted daily. The protocol also included a standardized set of blood chemistry tests on day 1, 2, 3 and 5 and a SARS-CoV-2 serology and SARS-CoV-2 PCR from nasal swabs on day 1 and 5.

### Study outcomes

The pre-specified primary outcome measure was number of days within 28 days after inclusion with a need for oxygen therapy to keep an oxygen saturation above 93%. Our hypothesis (h1) was that there is a significant difference in median time of oxygen- dependency between the groups. The endpoint was defined as the day after oxygen was last administered or the day after the patient was discharged from hospital in case the oxygen saturation was still not above 93% on discharge. Secondary outcomes were number of days before discharge from hospital, progression to need of HFNC or ventilator treatment at least one day after inclusion and all-cause mortality within 28 days (changed from the three month in the ClinicalTrial registration due to a maximum follow-up time of 28 days).

### Reasons for premature termination of the study

The study was initiated during the first wave of COVID-19 in which the incidence was low in the Skåne Region in the south of Sweden. Inclusion gained speed during the second wave, but at that time several high-quality studies were published, supporting that at the time of hospitalization when most patients have endogenous SARS-CoV2 antibodies, convalescence plasma is no longer beneficial [12, 13]. The study design, requiring oxygen need, excluded the few patients (usually elderly) who were admitted before day 7 when no antibodies could be expected. Moreover, after the start of vaccinations of elderly, mainly younger patients were hospitalized and usually late on day 10–14 when convalescent plasma had already been reported to be non-effective. We therefore decided to perform an interim analysis after 31 patients were included. The study was terminated after that since the data with long durations of oxygen need and a very high variation made it impossible to reach significant findings within the planned study.

### Statistics

Continuous variables were expressed as median with interquartile range (IQR) and categorical variables as numbers with proportion of total. Mann–Whitney-U test was used for differences between continuous variables and Fisher’s exact test for differences between categorical variables. Effect size was reported using the outcome measures median difference and odds ratio with 95% confidence interval. Statistical significance was defined as p < 0.05. Statistical analyses were performed using GraphPad Prism Release 7.

## Results

### Patients and randomization

**Fig. 1:**
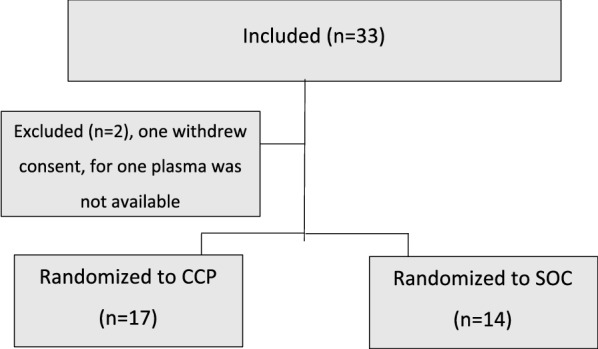
Flowchart of inclusion of cases of COVID-19

Between June 2020 and January 2021, 33 patients were included in the study. One patient in the SOC group withdrew consent immediately after randomization and one person in the CCP-group was excluded due to unavailability of ABO-compatible CCP. Thus 31 patients remained in the study and 14 were assigned to SOC and 17 to SOC and CCP (Fig. 1). Features of patients and concomitant medications are given in Table 1.

**Table 1:** Features of patients receiving SOC or CCP

	SOC (n = 14)	CCP (n = 17)
Demographics		
Age (years)^a^	65 (43–84)	80 (60–86)
Sex (male)^b^	8 (57)	11 (65)
BMI (kg/m^2^)^a^	31 (26–35)	28 (24–34)
Charlson comorbidity index^a^	1 (0–2)	1 (0–2)
Arterial hypertension^b^	6 (42)	7 (41)
Factors at inclusion		
Symptom duration (days)^a^	8 (5–11)	7 (5–9)
CRP (mg/L)^a^	100 (58–133)	72 (40–120)
Lymphocyte count (10^9^/L)^a^	0.8 (0.5–1.4)	0.9 (0.5–1.4)
Oxygen (L/min)^a^	2.5 (1–5)	2 (1.5–3)
Patients with SARS-CoV-2 antibodies at inclusion^a,c^	2 (18%)	1 (8%)
Treatment		
Betametason^b^	11 (79)	11 (65)
Remdesivir^b^	1 (7)	2 (12)
Intravenous antibiotics^b^	7 (50)	8 (53)
Anti-coagulants^b^	14 (100)	14 (82)

^a^Median (IQR). ^b^Numbers (%). ^c^Data available for 11 SOC-patients and 13 CCP-patients

One patient randomized to the CCP arm developed high fever within two hours after the first plasma administration, and received no further plasma units, but remained in the study. The remaining patients in this group received three doses of CCP. No other adverse events were recorded in any of the treatment arms. The study was ended after increasing evidence of futility of CCP in in-hospital treatment of COVID-19 and an interim-analysis indicating that there would be no difference in the primary outcome between the groups.

### Outcomes

The median time to primary endpoint among survivors was 7 days (IQR 5–9) for the SOC group and 11 days (IQR 6–15) for the CCP group (Table 2) (median difference −4, p = 0.4). Secondary outcomes are presented in Table 2. Three patients in the SOC group and two patients in the CCP group died (*p* = 0.64, OR 0.49, 95% CI 0.08–2.79). The casualties occurred on days 0, 10 and 28 in the SOC group and both on day 4 in the CCP group.

**Table 2:** Outcomes of patients receiving SOC or CCP

	SOC (n = 14)	CCP (n = 17)	Effect size	p-value
Time of oxygen treatment, days^e^	7 (5–9)^a^	11 (6–15)^a^	−4^c^	0.43
Length of stay, days	8 (6–10)^a^	13 (7–16)^a^	−5^c^	0.21
Progression to HFNC^f^	4/13 (31)^b^	4/16 (25)^b^	0.75 (0.18–3.18)^d^	> 0.99
Progression to ventilator treatment	1 (7)^b^	0^b^	0 (0–7.41)^d^	0.45
Death	3 (21)^b^	2 (12)^b^	0.49 (0.08–2.79)^d^	0.64

^a^Median (IQR). ^b^Numbers (%). ^c^Median difference. ^d^Odds ratio (CI).^e^For patients surviving 28-days. ^f^One patient in each group with ongoing HFNC on inclusion was excluded from the analysis

## Discussion

In this randomized controlled study of CCP treatment versus SOC, there was no significant difference between the groups in any primary or secondary outcome. We were thus unable to reject the null hypothesis. The results should be interpreted with caution due to the small sample size increasing the risk of a type 2 error. Our study aimed at including 100 patients but was prematurely terminated due to the growing body of evidence that CCP-treatment of COVID-19 in in-hospital patients is futile [12, 13].

There were many obstacles in conducting this study. Recruitment of plasma-donors was slow since our region had a low COVID-19 incidence during the spring of 2020 and there was a lack of standardized antibody tests. Inclusion of patients during the second wave (October to February) was mainly hampered due to language barriers and a high proportion of hospitalized COVID-19 patients with impaired cognitive abilities lacking the possibility to consent to the study protocol.

It has become increasingly clear that treatments targeting viral replication, including CCP, should be instituted early during COVID-19 [14]. Results from large well-performed randomized placebo-controlled studies were published or reported during the study period, all demonstrating that CCP has no beneficial effect at the time of hospitalization [12, 13, 15]. The inclusion criteria of our study requiring the need for oxygen therapy meant that patients admitted to hospital within a few days after the onset of symptoms could not be included at that point, since the need for oxygen therapy almost always occurred around day seven or later.

During the course of this study, CCP obtained for study-purposes was given outside the study as compassionate treatment to severely immune-suppressed individuals with COVID-19 who showed persistent viremia and/ or inability to develop antibodies. The results seem encouraging and we believe that a potential future role for CCP or monoclonal antibodies might be in the treatment of this group of patients [16–19].

## Limitations

The obvious limitation of this study is the premature termination resulting in a very small sample size. The intended sample size of 100 patients is also small compared to several published randomized studies on CCP and COVID-19. The endpoint was defined before the WHO-criteria for common outcome measure [20] which makes cross-study comparisons difficult.

## Supplementary Information

Additional file 1. SARS-CoV2 antibody detection and microneutralization assay.

## Data Availability

The datasets and detailed protocol during and/or analyzed during the current study are available from the corresponding author on reasonable request.

## Abbreviations

CCP: Convalescence plasma
COVID-19: Coronavirus disease-19
HFNC: High flow nasal cannula
IQR: Interquartile range
NIV: Non-invasive ventilation
NtAbs: Neutralizing antibodies
SARS-CoV2: Severe Acute Respiratory Syndrome Coronavirus 2
SOC: Standard of care
