# Supplementary material for: Convalescence plasma treatment of COVID-19: results from a prematurely terminated randomized controlled open-label study in Southern Sweden
Source: BMC Res Notes. 2021 Dec 4;14:440. doi: 10.1186/s13104-021-05847-7 (PMC8642769; doi:10.1186/s13104-021-05847-7)
Supplement: Supplementary file 1 — Additional file 1. SARS-CoV2 antibody detection and microneutralization assay. [file 13104_2021_5847_MOESM1_ESM.docx]

**Supplementary methods:**

IgG antibodies against SARS-CoV-2 spike protein in samples from all plasma donors were detected by in-house ELISA followed by the measurement of titers of **SARS-CoV2 neutralizing antibodies (NtAbs)** by a modified microneutralization assay on Vero E6 cells at the Department of Clinical Microbiology, Umeå University, Umeå, Sweden.

**SARS-CoV-2 S ELISA**

Clear flat-bottom Immuno Maxisorp 96-well plates (Thermo Scientific) were coated with 200 ng/well of purified SARS-CoV-2 spike (S) protein and the plates were incubated at +4°C overnight. The following day the wells were washed once with PBS-0.05% Tween 20 (PBS-T) and incubated for 1h at room temperature with blocking buffer (1% non-fat dry-milk in PBS-T). Duplicates of heat-inactivated (56°C, 30 min) plasma were diluted 1/50 in blocking buffer. For each plate, control serum samples from a highly anti-S IgG positive individual, and blank wells with blocking buffer alone was also included. Subsequently, the plates were incubated overnight at +4°C and the wells were washed four times with PBS-T. One hundred µl goat-anti human IgG horseradish peroxidase (HRP)-conjugated antibody (#H10007, Thermo Scientific) diluted 1/5000 in blocking buffer were added to each well and incubated for 1 h at room temperature (RT). The wells were then washed four times with PBS-T, followed by addition of 100µl/well of 1-Step Ultra TMB-ELISA (Thermo Scientific). The plate was incubated at RT for 15 min, and the reaction was stopped with 50 µl 2 M sulphuric acid per well. The plate was read spectrophotometrically at 450 nm with Tecan Sunrise. The final OD calculated as OD_405nm_(sample) – OD_405nm_ (blank well).

**Virus and cells**

VeroE6 cells were cultured in Dulbecco’s modified Eagle’s medium (DMEM, D5648 Sigma) supplemented with 5 % FBS (HyClone), 10 units/mL penicillin and 10 µg/ml streptomycin (PeSt, HyClone). SARS-CoV-2 (isolate SARS-CoV-2/01/human/2020/SWE, accession no/GeneBank no MT093571.1) was provided by the Public Health Agency of Sweden. The virus was propagated in VeroE6 cells for 72 h and the titer determined by plaque assay.

**Microneutralization assay**

Heat inactivated plasma samples were diluted 1:10 in virus solution (2x10^4^ PFU/ml in DMEM + PeSt) and then further five-fold serial diluted in same virus preparation. The mix was incubated at 37 ºC in 5 % CO_2_ for 30 min, before 50 µl was added as inoculum to VeroE6 cells seeded in 96-well plates (10 000 cells/well, Greiner CELLSTAR®) and incubated for another 2 h at 37 ºC in 5 % CO_2_ before the inoculum was removed and replaced with media containing DMEM + 2% FBS + PeSt. 8 h post infection the cells were first prefixed by removing 50 µl of media and adding 50 µl of 4 % formaldehyde for 10 min at RT, followed by fixation for 30 min in 4 % formaldehyde. Plates were washed with PBS and permeabilized with PBS + 0.5 % Triton X-100 + 20 mM glycine for 10 min at RT. Infected cells were stained 1 h with primary antibody (SARS-CoV; Sino Biological 40143-R001; 1:1000) followed by secondary antibody (A21206; Invitrogen; 1:1000) 30 min and DAPI staining (0.1 ug/mL in PBS) for 5 min. Number of infected cells were quantified using a TROPHOS Plate RUNNER HD® (TROPHOS SA, Marseille, France) and normalized to virus control incubated without plasma. IC_50_ values were calculated using Prism 8 (Graphpad software) using normalized data and a non-linear fit with variable slope.
